# Update to the study protocol: minimisation of dialysis risk in hospital patients with chronic kidney disease (MinDial): a multicentre, stepped-wedge, cluster-randomised controlled trial

**DOI:** 10.1186/s13063-024-08394-1

**Published:** 2024-09-04

**Authors:** D. Stelzer, H. Binder, M. Glattacker, E. Graf, M. Hahn, M. Hollenbeck, K. Kaier, B. Kowall, N. Kuklik, G. Metzner, N. Mueller, L. Seiler, S. Stolpe, C. Blume

**Affiliations:** 1 Institute of Medical Biometry and Statistics, Faculty of Medicine and Medical Center, University of Freiburg, Stefan-Meier-Str. 26, Freiburg, 79104 Germany; 2 Section of Health Care Research and Rehabilitation Research, Institute of Medical Biometry and Statistics, Faculty of Medicine and Medical Center, University of Freiburg, Hugstetter Straße 49, Freiburg, 79106 Germany; 3 Institute of Technical Chemistry, Leibniz University Hanover, Callinstraße 5, Hannover, 30167 Germany; 4 KfH Foundation for Preventive Medicine, Martin-Behaim-Straße 20, Neu-Isenburg, 63263 Germany; 5 Knappschaftskrankenhaus Bottrop GmbH, Academic Teaching Hospital of the University of Duisburg-Essen, Osterfelder Straße 157, Bottrop, 46242 Germany; 6 Knappschafts-Kliniken Service GmbH (KKSG), In der Schornau 23-25, Bochum, 44892 Germany; 7 Institute for Medical Informatics, Biometry and Epidemiology, University Hospital Essen, Hufelandstraße 55, Essen, 45147 Germany; 8 Centre for Clinical Trials Essen, University Hospital Essen, Hufelandstraße 55, Essen, 45122 Germany

**Keywords:** Chronic kidney disease (CKD), Kidney failure risk equation (KFRE), End-stage renal disease (ESRD), Estimated glomerular filtration rate (eGFR), Stepped-wedge design, Cluster-randomised trial (CRT)

## Abstract

This document reports a brief update to the previously published protocol of the MinDial study.

**Trial registration** German Clinical Trials Register DRKS00029691. Registered on 12 Sept. 2022, https://drks.de/search/en/trial/DRKS00029691.

## Update to the study protocol

The protocol of the MinDial study was previously published in *Trials* [1]. As stated in the subsection “Baseline data” of section “Participant timeline {13}”, the MinDial trial proceeds as follows:

“To document serum creatinine, KFRE score, blood pressure and HbA1c in the eCRF, all values are taken at a time interval around the date when the KFRE score is ≥ 15% (date of laboratory determination before 20 June 2023) or ≥ 9% (since 20 June 2023) for the first time in the current case.”

In June 2024, the study protocol was amended to be more specific on which of the measurements of the kidney failure risk equation (KFRE) score, serum creatinine and estimated glomerular filtration rate (eGFR) should be documented in the electronic case report form (eCRF), and to pre-specify the measurement designated as baseline. Accordingly, we would like to add the following as a supplement to the above paragraph:

“To be more specific, the KFRE score, serum creatinine and eGFR are documented at several time points:

- KFRE score:
  - measurement on the day of the current hospital stay on a peripheral ward on which the KFRE score is ≥ 15% or ≥ 9%, respectively, for the first time;
  - measurement closest to the inclusion date (baseline);
  - measurement closest to discharge;
- Serum creatinine: measurements on the same days as with the KFRE score;
- eGFR: measurements on the same days as with the KFRE score.

The measurements closest to the inclusion date are considered as the baseline and used for the evaluation.”

Apart from this, the study protocol remains unchanged.

## Data Availability

Not applicable.

## Abbreviations

eCRF: Electronic case report form
eGFR: Estimated glomerular filtration rate
KFRE: Kidney failure risk equation
